# Targeting Glycolysis in Macrophages Confers Protection Against Pancreatic Ductal Adenocarcinoma

**DOI:** 10.3390/ijms22126350

**Published:** 2021-06-14

**Authors:** Hweixian Leong Penny, Je Lin Sieow, Sin Yee Gun, Mai Chan Lau, Bernett Lee, Jasmine Tan, Cindy Phua, Florida Toh, Yvonne Nga, Wei Hseun Yeap, Baptiste Janela, Dilip Kumar, Hao Chen, Joe Yeong, Justin A. Kenkel, Angela Pang, Diana Lim, Han Chong Toh, Tony Lim Kiat Hon, Christopher I. Johnson, Hanif Javanmard Khameneh, Alessandra Mortellaro, Edgar G. Engleman, Olaf Rotzschke, Florent Ginhoux, Jean-Pierre Abastado, Jinmiao Chen, Siew Cheng Wong

**Affiliations:** 1 Singapore Immunology Network, A*STAR, Singapore, 8A Biomedical Grove Level 3 & 4 Immunos Building, Singapore 138648, Singapore; jelinlovelle@gmail.com (J.L.S.); gunsinyee@gmail.com (S.Y.G.); laumaichan@gmail.com (M.C.L.); Bernett_Lee@immunol.a-star.edu.sg (B.L.); jasmine_tan@immunol.a-star.edu.sg (J.T.); Cindy_phua@idlabs.a-star.edu.sg (C.P.); florida_toh@immunol.a-star.edu.sg (F.T.); Yvonnenga@gmail.com (Y.N.); Hseunhseun@gmail.com (W.H.Y.); Dilip_kumar@immunol.a-star.edu.sg (D.K.); hao.chen@lucence.com (H.C.); Yeongps@imcb.a-star.edu.sg (J.Y.); hanif.javanmard@irb.usi.ch (H.J.K.); mortellaro.alessandra@hsr.it (A.M.); Olaf_Rotzschke@immunol.a-star.edu.sg (O.R.); Florent_Ginhoux@immunol.a-star.edu.sg (F.G.); jp.abastado@gmail.com (J.-P.A.); Chen_Jinmiao@immunol.a-star.edu.sg (J.C.); 2 Skin Research Institute of Singapore (SRIS), 11 Mandalay Road, #17-01 Clinical Sciences Building, Singapore 308232, Singapore; Baptiste_Janela@sris.a-star.edu.sg; 3 Department of Pathology, Stanford University School of Medicine, 3373 Hillview Ave., Palo Alto, CA 94304, USA; justinkenkel1@gmail.com (J.A.K.); edengleman@stanford.edu (E.G.E.); 4 National University Cancer Institute Singapore, NUH Medical Centre (NUHMC) @ Levels 8-10, 5 Lower Kent Ridge Road, Singapore 119074, Singapore; angela_pang@nuhs.edu.sg; 5 Department of Pathology, National University Health System, National University Hospital, Lower Kent Ridge Road, 1 Main Building, Level 3, Singapore 119074, Singapore; diana_gz_lim@nuhs.edu.sg; 6 National Cancer Centre, 11 Hospital Crescent, Singapore 169610, Singapore; toh.han.chong@singhealth.com.sg; 7 Division of Pathology, Singapore General Hospital, 20 College Road, Academia, Level 7, Singapore 169856, Singapore; lim.kiat.hon@sgh.com.sg; 8 Perkin Elmer Australia, 530-540 Springvale Road, Melbourne VIC 3150, Australia; chris.johnson@perkinelmer.com

**Keywords:** pancreatic ductal adenocarcinoma, macrophage, immunometabolism, glycolysis

## Abstract

Inflammation in the tumor microenvironment has been shown to promote disease progression in pancreatic ductal adenocarcinoma (PDAC); however, the role of macrophage metabolism in promoting inflammation is unclear. Using an orthotopic mouse model of PDAC, we demonstrate that macrophages from tumor-bearing mice exhibit elevated glycolysis. Macrophage-specific deletion of Glucose Transporter 1 (GLUT1) significantly reduced tumor burden, which was accompanied by increased Natural Killer and CD8+ T cell activity and suppression of the NLRP3-IL1β inflammasome axis. Administration of mice with a GLUT1-specific inhibitor reduced tumor burden, comparable with gemcitabine, the current standard-of-care. In addition, we observe that intra-tumoral macrophages from human PDAC patients exhibit a pronounced glycolytic signature, which reliably predicts poor survival. Our data support a key role for macrophage metabolism in tumor immunity, which could be exploited to improve patient outcomes.

## 1. Introduction

Highly refractory to therapy, pancreatic ductal adenocarcinoma (PDAC) presents an intractable disease to patients, clinicians, and researchers alike. It is characterized by an overwhelming desmoplastic reaction, involving a combination of dense fibrosis and stromal infiltrate [1]. The latter is marked by progressive accumulation of immature myeloid cells and macrophages, establishing an ongoing, “smoldering” inflammation [2]. A number of studies have now established a clear link between inflammation and tumor etiology [3,4,5,6], confirming the importance of inflammation as a major driver of the neoplastic process.

Decades of work have focused on the metabolism of tumors. The preferential use of glycolysis even in the presence of oxygen, or Warburg metabolism, is a hallmark of tumor cell energetics [7,8]. Observed in over 90% of all cancers, it forms the basis for detection of tumor foci in the imaging modality ^18^FDG-PET [9,10]. Now, there is burgeoning interest in the field of immunometabolism [11,12] to define the requisite role of metabolic pathways in shaping immunity.

In the last decade, seminal work in the macrophage field highlight an intrinsic role of immune cell metabolism in the regulation of their effector phenotype [13,14]. Macrophage metabolism is involved in the various stages of tissue repair during the wound-healing response [15] and is also key to mediating the transcriptional and epigenetic adaptation of these cells during the pathogenesis of atherosclerosis [16]. More specifically, the tricarboxylic acid (TCA) cycle intermediate, succinate, was shown to direct IL1β production [17]. Similarly, blockage of glycolysis using 2-deoxyglucose (2-DG) inhibited macrophage activation in vitro [18,19] and suppressed inflammation in vivo [20]. While much of the field have described such advances in the inflammation and cardiometabolic therapeutic areas, a systematic evaluation of macrophage metabolism and its contribution in the cancer setting has not been addressed.

GLUT1 is a key isoform in the family of facilitative glucose transporters that mediate cellular glucose uptake. Comprehensive studies of this transporter in immune cells are ongoing in several laboratories, and our current understanding is that it is the predominant glucose transporter in macrophages [19,21,22,23,24]. In obese rats, GLUT1 is highly upregulated on macrophages in the inflamed adipose and liver tissue [19]. T_h_1, T_h_2, and T_h_17, but not T_Reg_ cells, express high surface levels of GLUT1 alongside elevated rates of glycolysis [25], indicating that GLUT1 is a key player in the metabolic program that drives pro-inflammatory phenotypes.

In a previous report, we showed that 2-DG blockade of glycolysis in PDAC tumor-conditioned macrophages in vitro reversed the pro-tumoral phenotype of these cells [26]. Here, we seek to extend these findings in an orthotopic model of PDAC and examine the effects of disrupting macrophage glycolysis in vivo on anti-tumor immunity.

## 2. Results

### 2.1. Macrophages from Tumor-Bearing Mice Are Highly Glycolytic

**Figure 1:**
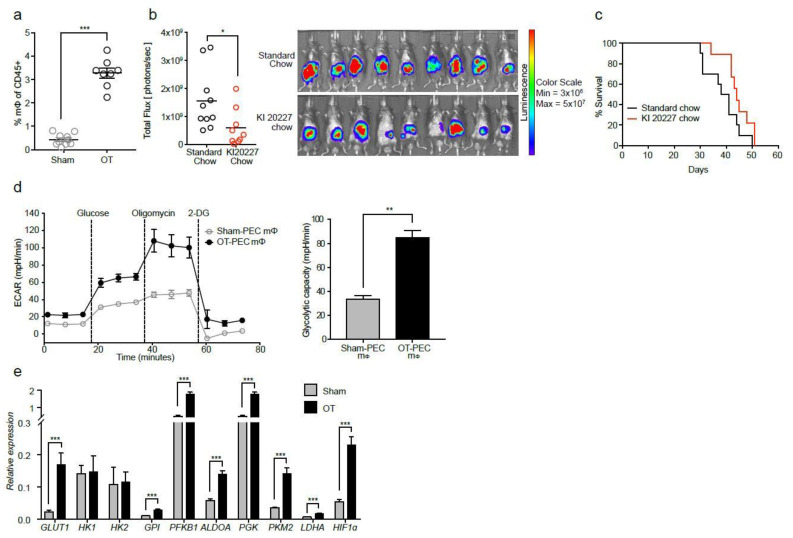
Macrophages from tumor-bearing mice are highly glycolytic. (**a**) Pancreata from Day 28 tumor-bearing orthotopically-transplanted (OT) mice were assessed for their myeloid cell populations by flow cytometry. Scatter plot of macrophages (CD45+ Lin- MHCIIlow-int CD24+ CD11b+ Ly6G- Ly6Clow F4/80+) that infiltrate the pancreas/tumor, as a percentage of total CD45+ cells, from OT (black) and age-matched sham controls (grey) (sham, *n* = 10; OT, *n* = 8, three independent experiments pooled). (**b**) OT mice were fed either KI20227 250 ppm (red) or standard rodent chow (black) beginning Day 1 of tumor cell transplantation. Representative scatter plot of the total flux on Day 26 as calculated from the BLI image shown (KI20227, *n* = 9; control, *n* = 10, two independent experiments). BLI images for all figures were acquired at 15-s exposure, and color scale set with lower limit at 3 × 10^6^ and upper limit at 5 × 10^7^ photons/sec. (**c**) Kaplan–Meier survival analysis of KI20227-fed mice (*n* = 9) compared with standard chow-fed mice (*n* = 10). (**d**) Sorted peritoneal (PEC) macrophages from OT and sham control mice were rested at 37 °C for 1 h and assessed for their glycolytic capacity in a live metabolic flux assay. Shown is a representative Seahorse trace of OT-PEC macrophages (black) compared with sham (grey) (**d**), and a bar graph of glycolytic capacity as calculated from the Seahorse trace (*n* = 3 mice pooled per group, five independent experiments). (**e**) PEC macrophages from OT and sham controls were assessed at the transcript level for GLUT1, HK1, HK2, GPI, PFKB1, ALDOA, PGK, PKM2, LDHA, and HIF1α (*n* = 3 mice pooled per group, three independent experiments). Data are means ± SEM, * *p* < 0.05; ** *p* < 0.01; *** *p* < 0.001 by unpaired student’s t-test with 95% confidence interval.

To generate an orthotopic model of PDAC, tumor cells derived from the KRAS^G12D/+^; *Trp53^R172H/+^;* PDX-cre (KPC) mice [27] were transplanted onto the pancreas. Prior to transplantation, tumor cells were stably transfected to overexpress luciferase to enable in vivo monitoring of tumor progression over time. Flow cytometry analysis, carried out 28 days after implanting the cells, confirmed a pervasive macrophage infiltration into the orthotopic tumors (OT) (3.2% of total CD45+ cells compared with 0.4% in sham-operated non-tumor bearing controls) (Figure 1a, Figure S1a). To assess the contribution of macrophages to tumor development, macrophages were depleted in vivo using KI 20227, a CSF1R-specific inhibitor. Notably, macrophage depletion resulted in a significant reduction in tumor burden. At Day 26 after tumor transplantation in vivo, bioluminescence imaging (BLI) of the tumor cells indicated significantly reduced signals compared with untreated controls (Figure 1b). Despite the delay in tumor kinetics, overall survival was unchanged (Figure 1c), indicating that this strategy was not sufficient to increase survival in PDAC, in contrast to previous observations in models of brain [28] and mammary carcinomas [29].

To determine if the metabolic state of macrophages influenced tumor progression, we assessed glycolysis using a Seahorse live metabolic flux assay (Figure 1d, left panel). Macrophages from peritoneal exudate cells (PEC) in OT mice exhibited nearly three-fold higher rates of glycolysis compared with sham controls (Figure 1d, right panel). qPCR analysis further revealed upregulated transcript expression of the key glycolytic enzymes GLUT1, glucose-6-phosphate isomerase (*GPI*), phosphofructokinase (*PFKB1*), fructose biphosphate aldolase A (*ALDOA*), phosphoglycerate kinase (*PGK*), pyruvate kinase muscle isozyme M2 (*PKM2*), lactate dehydrogenase A (*LDHA*), and hypoxia-inducible factor 1-alpha (HIF1α), a key regulator in hypoxia responses (Figure 1e). Notably, we saw no evidence of mitochondrial dysfunction in these macrophages. The transcript levels of the TCA cycle enzymes pyruvate dehydrogenase kinase 1 (*PDK1*) and succinate dehydrogenase complex iron sulfur subunit B (*SDHB*) were significantly upregulated, isocitrate dehydrogenase 1 (*IDH1*) was downregulated, while citrate synthase (*CS*), fumarate hydratase 1 (*FH1*) and malate dehydrogenase 1 (*MDH1*) were not significantly increased (Figure S1b). Taken together, we show that macrophages from OT mice exhibited a strongly glycolytic profile with apparently normal mitochondrial function.

### 2.2. Macrophage-Specific Deletion of GLUT1 Confers Resistance to Tumor Growth

**Figure 2:**
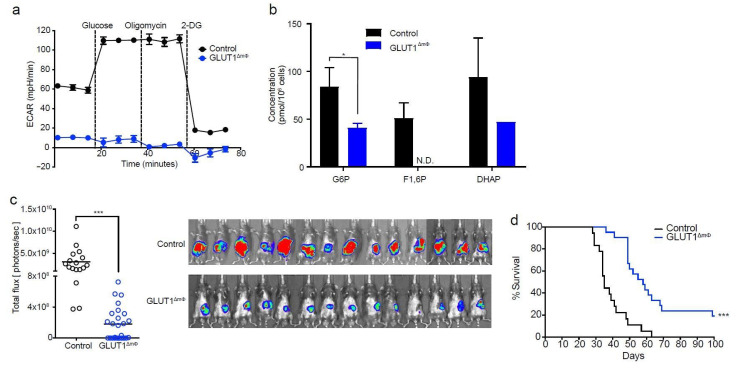
**Macrophage-specific deletion of GLUT1 confers resistance to tumor growth.** (**a**) Sorted PEC macrophages from control (LysM-cre and/or GLUT1fl/fl alone) and GLUT1ΔmΦ mice were rested at 37 °C for 1 h and assessed for their glycolytic capacity in a live metabolic flux assay. Shown is a representative Seahorse trace of control (black) compared with GLUT1ΔmΦ (blue) (*n* = 3 mice pooled per group, three independent experiments). (**b**) Ratio of area under mass peak of each metabolite in the glycolysis pathway derived from metabolomics profiling of control (black) and GLUT1ΔmΦ (blue) PEC macrophages (control, *n* = 3; GLUT1ΔmΦ, *n* = 4, *n* = 3 mice pooled per sample). (**c**) Orthotopic tumors from control and GLUT1ΔmΦ mice were monitored in vivo over time using BLI. Shown is a representative scatter plot of the total flux on Day 28 as calculated from the BLI images (control, *n* = 17; GLUT1ΔmΦ, *n* = 25, pooled from four independent experiments). (**d**) Shown is a Kaplan–Meier survival analysis of GLUT1ΔmΦ mice (*n* = 21) compared with controls (*n* = 18), pooled from two independent experiments. (Data are means ± SEM, * *p* < 0.05; *** *p* < 0.001 by unpaired student’s t-test with 95% confidence interval.

To assess the role of macrophage glycolysis in disease outcome, we bred GLUT1^fl/fl^ mice with LysM-Cre mice, to generate mice with a macrophage-specific deletion of GLUT1 (henceforth called GLUT1^ΔmΦ^). Extracellular flux analysis indicated that macrophages from GLUT1^ΔmΦ^ mice were completely lacking the functional capacity for glycolysis (Figure 2a). Extensive metabolomics analysis confirmed that key glycolysis intermediates, such as fructose 1,6-biphosphate (F1,6P) were almost undetectable compared with controls (Figure 2b, Figure S2a).

Despite the loss of glycolytic function, GLUT1^ΔmΦ^ macrophages were able to perform oxidative phosphorylation at levels comparable with control macrophages (Figure S2a). Pancreatic macrophage cell numbers, as a proportion of CD45+ cells remained unchanged in GLUT1^ΔmΦ^ compared with control tumor-bearing mice, indicating that cell viability was not affected (Figure S2b). Importantly, however, tumor cells transplanted into GLUT1^ΔmΦ^ mice resulted in a dramatic reduction of tumor burden at all time points compared with controls. BLI imaging performed on Day 28 revealed a significantly reduced signal in each of the GLUT1^ΔmΦ^ mice compared to the control group (Figure 2c). In contrast to KI20227-treated mice, GLUT1^ΔmΦ^ mice also displayed significantly improved overall survival (Figure 2d). Given the beneficial outcome of macrophage-specific ablation of glycolysis in the tumor response, we next focused our efforts on elucidating the downstream cellular changes mediating immune effector function.

### 2.3. The pro-Inflammatory NLRP3-IL1β Axis Is Suppressed in GLUT1^ΔmΦ^ Macrophages

**Figure 3:**
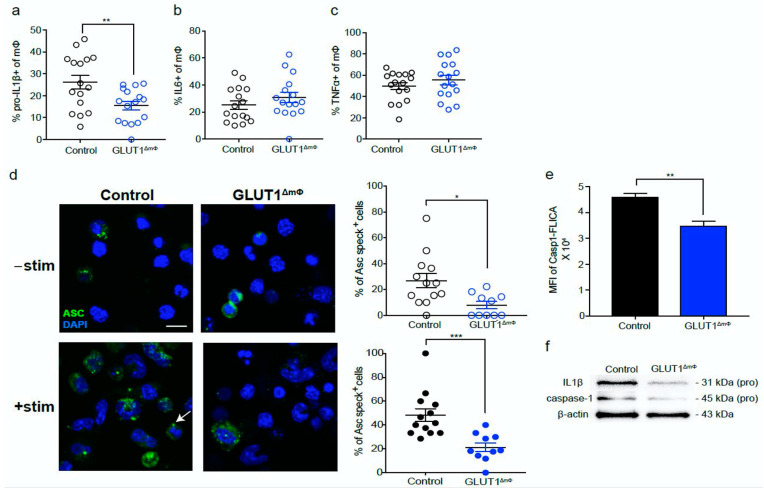
The pro-inflammatory NLRP3-IL1β axis is suppressed in GLUT1ΔmΦ macrophages. (**a**–**c**) Intracellular flow cytometry (ICFC) of pro-inflammatory cytokines was performed on control (black) and GLUT1ΔmΦ (blue) pancreata cell suspensions restimulated with 100 ng/mL LPS for 4 h. Shown are scatter plots of the % pro-IL1β+ (**a**), % IL6+ (**b**), % TNFα+ (**c**) of total macrophages (control, *n* = 16; GLUT1ΔmΦ, *n* = 16, pooled from three independent experiments). Representative flow plots are shown in Supplementary Figure S3b. (**d**) Immunofluorescence staining of ASC specks was performed in FACS-sorted control (black) and GLUT1ΔmΦ (blue) PEC macrophages stimulated with 1 μg/mL of LPS for 3 h, and 10 μM of nigericin for a further 30 min (+stim, bottom row) or without (-stim, top row). Shown are representative confocal images with nuclei (blue) and ASC (green) overlaid. Each ASC speck is identified as a dot-like perinuclear stain as shown by the representative white arrow in the bottom left image. Magnification bar = 10 μM. Scatter plots shown are the quantification of % ASC speck+ cells for every field of view in unstimulated (open circles) and stimulated cells (filled circles) (*n* = 3 mice pooled per group, two independent experiments). (**e**) FACS-sorted control (black) and GLUT1ΔmΦ (blue) PEC macrophages were stained with Caspase-1-FAM-FLICA to assess caspase-1 activity by flow cytometry. Shown is a representative bar graph of the MFI of caspase-1 (*n* = 3 mice pooled per group, two independent experiments). (**f**) Representative immunoblot of sorted control and GLUT1ΔmΦ pancreatic tumor macrophage cell lysates probed for pro-IL1β and pro-caspase 1 (*n* = 6–8 mice pooled per group, two independent experiments). Data are means ± SEM, * *p* < 0.05; ** *p* < 0.01; *** *p* < 0.001; n.s. not significant, by unpaired student’s t-test with 95% confidence interval.

NLRP3 signaling has been shown to promote tumor growth in PDAC [30]; therefore, we hypothesized that the NLRP3-IL1β inflammasome may be activated in the orthotopic model and may contribute to the pro-tumoral immune response. Consistent with this hypothesis, we observed that PEC macrophages from orthotopically-transplanted mice expressed higher pro-IL1β compared with sham controls (Figure S3a). In line with this, we observed that the pro-IL1β expression in GLUT1^ΔmΦ^ macrophages was significantly reduced (Figure 3a), while no differences in IL-6 and TNFα production were observed compared with controls (Figure 3b,c). We further assessed key measures of NLRP3 activation in GLUT1^ΔmΦ^ macrophages, such as assembly of apoptosis-associated speck-like protein containing a CARD (ASC) specks, and the activity of caspase 1, the catalytic enzyme that cleaves pro-IL1β to the active form of IL1β. The proportion of ASC speck-positive GLUT1^ΔmΦ^ macrophages was reduced by > 3-fold compared with controls (7.91% vs. 25.0%). Even upon LPS and nigericin stimulation, two agents are known to induce NLRP3 inflammasome activation, the proportion of ASC speck-positive GLUT1^ΔmΦ^ macrophages remained downregulated compared with controls (21.2% vs. 48.3%) (Figure 3d). The downregulation of caspase 1 activity was confirmed by a Caspase 1 Fluorescein (FLICA) assay (Figure 3e), while the reduced protein expression of IL1β and caspase 1 in GLUT1^ΔmΦ^ macrophages could be established by immunoblotting (Figure 3f). These results support the notion that the NLRP3-IL1β inflammasome pathway was suppressed in GLUT1^ΔmΦ^ macrophages.

### 2.4. NK Cells and CTLs Mediate Anti-Tumor Immunity in GLUT1^ΔmΦ^ Mice

**Figure 4:**
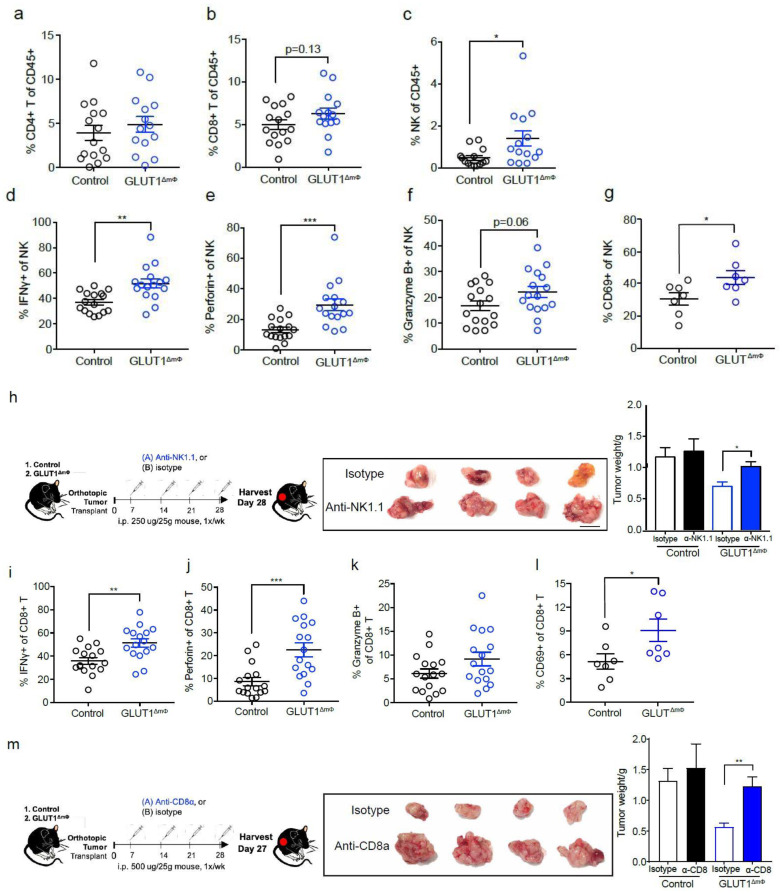

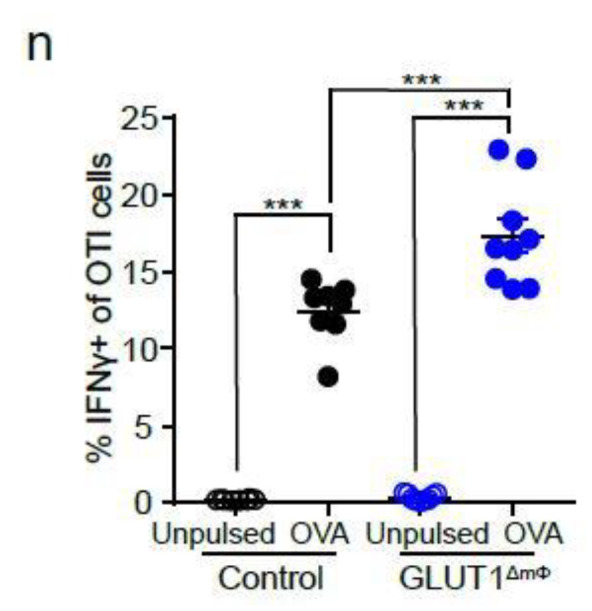
NK cells and CTLs mediate anti-tumor immunity in GLUT1ΔmΦ mice. (**a**–**g**, **i**–**l**) Flow cytometry was performed in. control and GLUT1ΔmΦ mice to assess NK and CTL populations. Flow cytometry gating strategy shown in Supplementary Figure S4b. Shown are scatter plots of the % CD4+ T cells (**a**), % CD8+ T cells (**b**), and % NK cells (**c**) of the total CD45+ population in the pancreata of control (black) and GLUT1ΔmΦ (blue) mice (*n* =14 mice per group, pooled from four independent experiments). Shown are scatter plots of % IFNγ+ (**d**), % Perforin+ (**e**), % Granzyme B+ (**f**) (*n* = 16 mice per group, pooled from three independent experiments, representative flow plots are shown in Supplementary Figure S4c) and % CD69+ (**g**) (*n* = 6 mice per group) of total NK cells in the pancreata of control (black) and GLUT1ΔmΦ (blue) mice. (**h**) NK cells were depleted in vivo by injecting 250 μg of either isotype (open bars) or anti-NK1.1 (filled bars) antibody i.p. per mouse, once a week. Shown are bar graphs of the tumor weight measured at Day 28 of control and GLUT1ΔmΦ mice (*n* = 5 mice per group, representative of two independent experiments). (**i**–**l**) Shown are scatter plots of % IFNγ+ (**i**), % Perforin+ (**j**), % Granzyme B+ (**k**) (*n* = 16 mice per group, pooled from three independent experiments) and % CD69+ (**l**) (*n* = 6 mice per group_of total CD8+ T cells in the pancreata of control (black) and GLUT1ΔmΦ (blue) mice. (**m**) CD8+ T cells are depleted in vivo by injecting 500μg of either isotype (open bars) or anti-CD8α (filled bars) antibody i.p. per mouse, once a week. Shown are bar graphs of the tumor weight measured at Day 27 of control and GLUT1ΔmΦ mice (at least *n* = 5 mice per group). Data are means ± SEM, * *p* < 0.05; ** *p* < 0.01; *** *p* < 0.001 by unpaired student’s t-test with 95% confidence interval. (**n**) Scatter plot of % IFNγ+ CD8+ OTI cells after co-culture with either unpulsed or OVA-pulsed macrophages isolated from the pancreata of control (black) and GLUT1ΔmΦ (blue) mice (*n* ≥ 8 mice per group). Data are means ± SEM, *** *p* < 0.001 by one-way ANOVA with Bonferroni post-hoc test.

We next considered whether the observed resistance to tumor growth in GLUT1^ΔmΦ^ mice might involve changes in the anti-tumor lymphocyte response. Following an extensive characterization of the lymphoid populations in these mice, we observed no differences in the proportions of CD4+ T cells (Figure 4a) and Th17 cells (Figure S4) between GLUT1^ΔmΦ^ mice and controls. While the proportion of CD8+ cytotoxic T lymphocytes (CTLs) trended upwards in GLUT1^ΔmΦ^ (*p*-value = 0.13), they did not reach significance (Figure 4b). However, NK cell numbers were consistently upregulated in GLUT1^ΔmΦ^ mice compared with controls (Figure 4c), and expressed elevated amounts of IFNγ, perforin, and granzyme B, indicating enhanced cytotoxic function (Figure 4d–f). Upregulated expression of CD69 further demonstrated that NK cells in GLUT1^ΔmΦ^ mice were more activated (Figure 4g). To investigate whether NK cells were required for mediating anti-tumor immunity, they were depleted from GLUT1^ΔmΦ^ mice with anti-NK1.1 (PK136) antibody (Figure 4h). Compared with isotype controls, NK cell-depleted GLUT1^ΔmΦ^ mice had a significant enlargement in their tumor sizes (Figure 4h), indicating that NK cells were crucial in controlling tumor growth in GLUT1^ΔmΦ^ mice.

Although numbers of CTLs were not significantly changed, similar to NK cells, they were also found to upregulate expression of IFNγ, perforin, and granzyme B in GLUT1^ΔmΦ^ mice (Figure 4i–k). Furthermore, a higher fraction of these CTLs expressed CD69 (Figure 4l), indicating the presence of a more activated CTL population in GLUT1^ΔmΦ^ mice than control mice. Depleting CTLs with anti-CD8α (YTS 169.4) antibody in GLUT1^ΔmΦ^ mice resulted in significantly larger tumors compared to the isotype control treated mice (Figure 4m), indicating that CTLs are also key to controlling tumor growth in GLUT1^ΔmΦ^ mice. Taken together, we show that protection from tumor growth in GLUT1^ΔmΦ^ mice is mediated in part, by the classical effectors of anti-tumor immunity—NK cells and CTLs.

To determine if the poor CTL response in WT orthotopically-transplanted mice is attributed to suboptimal antigen presentation by the glycolytic macrophages, we isolated peritoneal macrophages on Day 28 from control and GLUT1^ΔmΦ^ orthotopically-transplanted mice. After in vitro loading with OVA (SIINFEKL) peptide, these macrophages were co-cultured with OVA-specific (OTI) T cells for 6 hr before IFNγ production by OTI T cells was assessed using flow cytometry. Without OVA peptide pulsing, both control and GLUT1^ΔmΦ^ macrophages induced a similar level of IFNγ secretion in OTI T cells. However, in the presence of OVA peptide, GLUT1^ΔmΦ^ macrophages stimulated OTI T cells to produce significantly more IFNγ than control macrophages (Figure 4n), suggesting that the inability to utilize glycolysis may be enhancing their antigen presentation capacity.

### 2.5. The GLUT1 Inhibitor WZB117 Attenuates Tumor Burden In Vivo

Although GLUT1 is a widely-expressed glucose transporter present on several types of carcinomas [31,32,33,34], a lack of specific and potent inhibitors have hindered its potential as a therapeutic target. New-generation GLUT1 inhibitors, including WZB117, have been synthesized that overcome these limitations [35]. Given that genetic ablation of GLUT1 in macrophages was successful in mitigating tumor burden in this orthotopic transplant model, we hypothesized that WZB117 treatment might result in a similar effect. WZB117 treatment (50 and 100 µM) was able to transiently block glycolysis of orthotopically-transplanted PEC macrophages in vitro for approximately 2–3 h (Figure S5a). As expected, WT PEC macrophages, which were previously shown to express low levels of GLUT1 (Figure 1e), were unresponsive to WZB117. In a pilot experiment with mice bearing 2-week-old orthotopic tumors, we could establish that a dose of 40 mg/kg delivered i.p. daily over a period of 7 days was effective in blocking glycolysis in vivo (Figure S5b). Doses up to 50 mg/kg i.p. daily for 7 days were well-tolerated in mice (mice did not display signs of inanition; data not shown).

**Figure 5:**
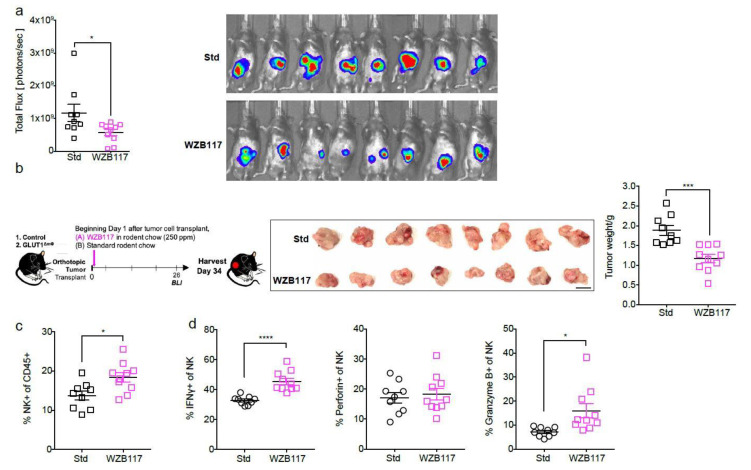

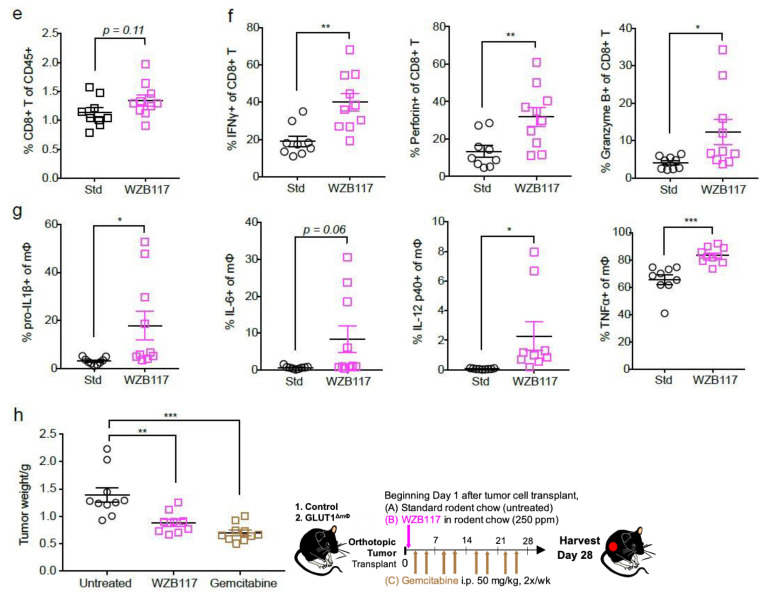
The GLUT1 inhibitor WZB117 attenuates tumor burden in vivo. WZB117 (250 ppm) was fed to mice beginning Day 1 of orthotopic transplantation of tumor cells. Disease progression was monitored in vivo, and immune parameters were assessed in the pancreatic tumors. (**a**) Scatter plot of the total flux on Day 26 of mice fed with standard (“Std”) rodent chow (black) (*n* = 9) or WZB117-incorporated chow (magenta) (*n* = 10) as calculated from BLI images. (**b**) The same mice from (**a**) were euthanized and shown is a scatter plot of their tumors weighed at Day 34. Tumor images are shown on the right hand side. Scale bar = 1 cm. (**c**–**g**) Shown are scatter plots of the % NK of total CD45+ cells (**c**), % IFNγ+, % Perforin+, and % Granzyme B+ of NK cells (d), % CD8+ T of total CD45+ cells (**e**), % IFNγ+, % Perforin+, and % Granzyme B+ of CD8+ T cells (**f**), % pro-IL1β+, % IL6+, % IL12p40+, and % TNFα+ (g) of total macrophages. Data representative of two independent experiments, with *n* = 9–10 mice per group. (**h**) WZB117 was compared head to-head with standard-of-care gemcitabine. Gemcitabine was injected 50 mg/kg i.p., twice a week, beginning the first week. Shown is a scatter plot of tumors from mice fed standard rodent chow (“untreated”) (black), WZB117-incorporated chow (magenta), or treated with gemcitabine (brown) weighed at Day 28. Data representative of two independent experiments, *n* = 10 mice per group. Data are means ± SEM, * *p* < 0.05; ** *p* < 0.01; *** *p* < 0.001 by unpaired student’s t-test with 95% confidence interval.

To test whether the WZB117 treatment could also improve disease outcome in our mouse model, orthotopically-transplanted mice were fed WZB117 at 250 ppm (equivalent to a 50 mg/kg daily dose) beginning Day 1 of tumor cell transplantation. By Day 26, BLI indicated a reduced tumor burden in WZB117-fed mice compared with mice fed standard rodent chow (Figure 5a). When euthanized at Day 34, a reduction in tumor weight was confirmed in WZB117-fed animals compared with controls (Figure 5b). The proportion of NK cells was also increased in WZB117-fed mice compared with standard chow-fed mice (Figure 5c), and these cells displayed increased expression of IFNγ and granzyme B (Figure 5d). The relative fraction of CD8+ T cells again trended upwards (Figure 5e), and the cells had significantly increased expression of IFNγ, perforin, and Granzyme B (Figure 5f). While these results mirrored our observations in GLUT1^ΔmΦ^ mice (Figure 4b–f,h–j), we noted significantly higher, not lower, levels of pro-IL1β, IL12p40, and TNFα proinflammatory cytokines in WZB117-fed mice (Figure 5g), which differ from the observations in GLUT1^ΔmΦ^ mice.

Using our orthotopic model of PDAC, treatment with WZB117 was then compared head-to-head with gemcitabine, a synthetic pyrimidine nucleoside prodrug representing the current standard-of-care chemotherapy for PDAC patients. Low-dose gemcitabine (50 mg/kg) administered via i.p. injection twice weekly in a spontaneous model of PDAC was previously shown to significantly increase median survival compared with placebo controls [36]. Using the same dosing regimen, beginning Day 1 of orthotopic tumor transplantation to synchronize with WZB117 treatment, we observed a comparable reduction in tumor size in WZB117- and gemcitabine-treated mice versus untreated mice (Figure 5h). These results highlight the potential of GLUT1 inhibitors, such as WZB117, as a therapeutic strategy to improve disease outcomes.

### 2.6. The Macrophage Glycolytic Signature Predicts Poor Survival in Human PDAC

**Figure 6:**
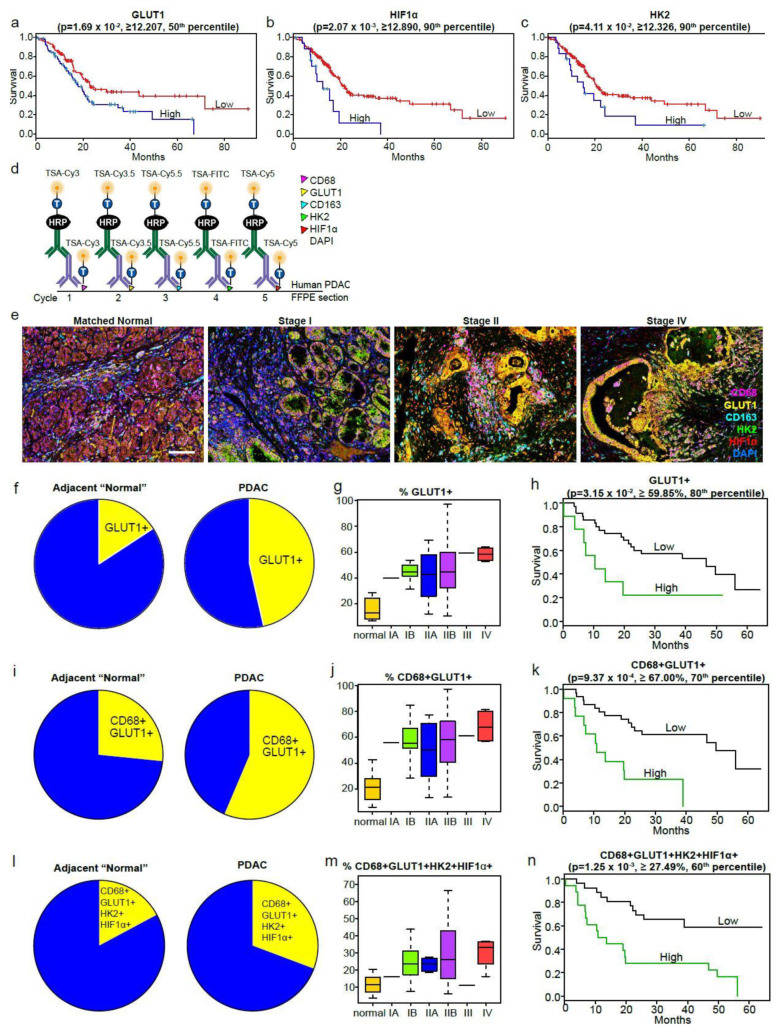
The macrophage glycolytic signature is a predictor of poor survival in human PDAC. (**a**–**c**) Gene expression and clinical survival data was extracted from the TCGA cohort, a publicly available dataset. Expression of GLUT1 (**a**), HIF1α (**b**), and HK2 (**c**) were thresholded at various percentiles and the binary states (patients above the cut-off percentile was defined as “high”, and those below defined as “low”) for each marker were used in a log-rank survival analysis. Shown are Kaplan-Meier survival curves of GLUT1 (**a**), HIF1α (**b**), and HK2 (**c**) for which the percentile cut-offs yielded significant difference between the high and low patient populations. (**d**) Schematic of the sequential, multiplex, immunofluorescence staining protocol of myeloid markers (CD68 and CD163) and metabolic markers (GLUT1, HIF1α, and HK2) used to stain FFPE sections from multi-center patient cohorts. (**e**) Shown are representative images from matched normal and patients at different stages of PDAC (Stage III not shown, as we had only one stage III patient among our cohorts). CD68 (magenta), GLUT1 (yellow), CD163 (aqua), HK2 (green), HIF1α (red), DAPI (blue). Scale bar = 100 µM. (**f**–**m**) The expression of GLUT1, HK2, and HIF1α on CD68+ macrophages was objectively quantified. CD68+ cells co-expressing each glycolytic marker (called “subsets”) were defined as a percentage of total CD68+ cells in individual patients. Shown are pie charts of the mean of the %GLUT1+ (**f**) in total cells, % CD68+GLUT1+ subset (**i**) and % CD68+ GLUT1+HK2+HIF1α+ (**l**) in adjacent “normal” versus PDAC (collectively) as a representation of total CD68+ cells. Shown are box plots of the mean ± s.d. of the %GLUT1 (**g**), % CD68+GLUT1+ subset (**j**) and % CD68+ GLUT1+HK2+HIF1α+ (**m**) across the different stages of disease compared with adjacent “normal” controls. Similar to Figure 6a, the percentage composition of the GLUT1+ (**h**), CD68+GLUT1+ subset (**k**) and CD68+ GLUT1+HK2+HIF1α+ subset (**n**) were thresholded at various percentages and the binary states (patients above the cut-off percentage was defined as “high”, and those below defined as “low”) were used in a log-rank survival analysis. Shown are Kaplan-Meier survival curves for the two subsets in which the percentage cut-offs yielded significant difference between the high and low patient populations.

To validate our observations in human PDAC, we examined the expression of selected glycolytic markers, including GLUT1, Hexokinase 2 (HK2,) and HIF1α, using publicly available PDAC patient data. We extracted gene expression and clinical survival data of PDAC patients from The Cancer Genome Atlas (TCGA) and the Queensland Centre for Medical Genomics (QCMG) cohorts. GLUT1, HK2, and HIF1α were thresholded at the indicated percentages (GLUT1,≥12.207%; HK2, ≥12.89%; HIF1α, ≥12.326%) and the binary states (patients above the cut-off percentile defined as “high”, and those below defined as “low”) for each marker were used in a log-rank survival analysis. We observed that GLUT1^high^, HK2^high^, and HIF1α^high^ patients from the TCGA cohort exhibited poorer overall survival (GLUT1, *p* = 1.69 × 10^−2^; HK2, *p* = 2.07 × 10^−3^; HIF1α, *p* = 4.11 × 10^−2^) (Figure 6a–c) and disease-free survival (Figure S6a). Similar poorer overall survival curves were observed using patient data from the QCMG cohort stratified using the same method (Figure S6b).

To assess whether the expression of these glycolytic markers was an important prognosticator of disease outcome, formalin-fixed paraffin-embedded (FFPE) sections of PDAC patients from three different centers were obtained. The FFPE sections were stained in a sequential, multiplex, immunofluorescence staining protocol (Figure 6d) to enable visualization and quantification of the following six markers: GLUT1, HK2, HIF1α (glycolytic markers), CD68 (a macrophage marker), CD163 (a tumor-associated, or M2-associated marker), and DAPI (nuclear marker). Images representative of patients in each tumor stage are shown (Figure 6e, Stage III not shown as only one Stage III patient was present in our cohorts). We objectively quantified the expression of GLUT1 on total cells as well as GLUT1, HK2, and HIF1α on CD68+ macrophages. GLUT1+ cells were defined as a percentage of total DAPI+ cells in each patient whereas each glycolytic marker (called “subsets”, e.g., CD68+ GLUT1+ subset) expressed on macrophages were defined as a percentage of total CD68+ cells in each patient. Individual cohorts were separately analyzed first, before combining into a single patient cohort to check that the three cohorts displayed similar trends.

The analysis revealed a three-fold higher percentage of GLUT1+ cells, in PDAC patients (46.4%) compared to adjacent “normal” controls (15.8%) (Figure 6f). Although GLUT1 expression did not correlate with disease stage (I-IV) (Figure 6g), GLUT1^high^ patients (with ≥59.85% of GLUT1+ cells) exhibited poorer survival outcomes (Figure 6h) compared to GLUT1^low^ patients. To further determine whether macrophage glycolysis correlated with clinical survival, we performed a deeper analysis focusing on glycolytic marker expression on macrophages. A Kruskal–Wallis Rank Sum test revealed four subsets that were not confounded by race or gender, and these were selected for downstream analysis and correlation to clinical parameters. Consistent with our hypothesis that macrophage glycolysis is key in determining PDAC disease outcome, two of the four subsets correlated with disease outcome—(1) CD68+ GLUT1+ and (2) CD68+ GLUT1+ HK2+ HIF1α+ (“3-marker+”) subsets. The percentage of the CD68+ GLUT1+ subset was more than two-fold higher in PDAC patients (56.4%) compared with matched normal controls (26.6%) (Figure 6i). The 3-marker+ subset exhibited smaller, but also significant differences between PDAC patients (30.7%) and matched normal controls (17.2%) (Figure 6l). Although there was no correlation with disease stage (I-IV) for either subset (CD68+ GLUT1+, *p* = 0.80 (Figure 6j); 3-marker+, *p* = 0.68 (Figure 6m)), we observed that CD68+ GLUT1^high^ (≥67.00%) (Figure 6k) or the 3-marker+^high^ patients (≥27.49%) (Figure 6n) exhibited much poorer survival compared to CD68+ GLUT1^low^ and 3-marker^low^ patients. Both CD68+ GLUT1+ (hazard ratio (HR) = 3.75) and 3-marker+ (HR = 3.49) have higher hazard ratios than GLUT1+ (HR = 2.56). This clearly demonstrates that glycolytic macrophages could predict PDAC survival more accurately than GLUT1 expression on all cells (Table S3). Taken together, these results in human PDAC validate our findings in the mouse model—that the macrophage glycolytic signature is a potent predictor of survival in PDAC.

## 3. Discussion

The connection between dysregulated glucose metabolism and tumor etiology is well supported by epidemiological evidence. Indeed, 80% of all PDAC patients are diabetic, and 15% of patients exhibit new-onset diabetes at the point of diagnosis [37]. Indeed, Ying and colleagues demonstrate that oncogenic KRAS directly promotes anabolic glucose metabolism in an inducible KRAS*^G12D^*-driven PDAC mouse model [38]. Previously, much of the focus has been on tumor cell metabolism itself with little known about the metabolic pathways utilized in the stroma. However, recent studies investigating the role of immunometabolism in disease pathogenesis have revealed the importance of this previously unappreciated field [13,14,26,39,40,41,42,43,44].

Here, we demonstrate that macrophages from an orthotopic model of PDAC are highly glycolytic. This metabolic profile is consistent with a tumor microenvironment in which inflammation promotes tumor growth, and is in line with previous reports describing enhanced glycolysis in LPS-activated macrophages [19,45]. Glycolysis, while not the most effective way to generate ATP (two ATP molecules from one molecule of glucose), can be activated rapidly via the induction of glycolytic enzymes. This is in contrast to oxidative phosphorylation (OXPHOS), the engagement of which is more time-consuming and more complex as it requires mitochondrial biogenesis [11,46]. The metabolic shift to glycolysis in the macrophages seen in our orthotopic transplant model may enable these cells to generate rapid ATP production, and stimulate pro-inflammatory cytokine production. Indeed, previous reports have shown that the induction of the glycolytic enzyme PKM2 in LPS-activated macrophages promoted the expression of HIF1α-dependent genes, such as IL1β [46,47]. PKM2 has also been shown to have a pro-inflammatory role in human atherosclerotic plaques [48]. Another glycolytic enzyme, HK1, was found to interact with the NLRP3 inflammasome at the outer mitochondrial membrane, enabling NLRP3 activation [49]. In support of the link between macrophage glycolysis and inflammation, we observed upregulation of PKM2 in macrophages of the orthotopic-transplant model compared with sham controls, although HK1 expression was unchanged.

Metabolomics profiling revealed that GLUT1^ΔmΦ^ macrophages, following genetic ablation of the major glucose transporter GLUT1, have reduced amount of glycolysis intermediates confirming a shutdown in the glycolysis pathway. Given that the pancreatic macrophages in GLUT1^ΔmΦ^ orthotopically-transplanted mice appeared to have normal mitochondrial function, these macrophages could have switched to utilizing an alternative energy source. Fatty acids (FA) and glutamine are two major energy fuels, which can be metabolized to drive the TCA cycle and ultimately support mitochondrial function. Our results are consistent with a report by Freemerman and colleagues, showing that bone marrow-derived macrophages (BMDMs) from a murine model of myeloid-specific GLUT1 deletion generated similar to ours, exhibited reduced glycolysis [50]. However, in contrast to that report, we observed functional OXPHOS in the macrophages we assayed. This may be due to differences in (1) the type of macrophages each laboratory evaluated, and (2) the disease context in which the macrophages were being assessed. We assayed freshly-isolated PECs derived from an orthotopically-transplanted tumor model, while Freemerman and colleagues assayed ex-vivo cultured BMDMs and macrophages isolated from adipose tissue in models of diet-induced obesity and atherosclerosis [50]. Of note, GLUT1-ablated BMDM in their model showed evidence of substrate switch, utilizing FA and glutamine metabolism in the absence of GLUT1-mediated glycolysis and pentose phosphate pathway (PPP), which interestingly translated to a mixed inflammatory phenotype [50].

Indeed, several recent reports have shown that long-chain FAs, such as palmitate, induce IL1β signaling and mediate the pro-inflammatory response in type 2 diabetes [51]. Abnormal accumulation of fatty acids and lipoproteins in macrophages was observed to correlate with foam cell formation and with the etiology of inflammatory pathologies, such as atherosclerotic plaques [52,53,54]. Moreover, a build-up of unsaturated fatty acids, such as oleic acid and linoleic acid, was shown to stimulate the production of IL1α in foam cells, promoting inflammation in vivo [55]. Conversely, glutamine uptake by in vitro stimulated macrophages exhibited enhanced production of inflammatory cytokines such as IL1 [56], IL6 [57], and TNFα [58,59]. The accumulation of succinate, a downstream intermediate of glutamine catabolism, was shown to promote a pro-inflammatory response in tumor-associated macrophages [60]. However, recent cumulative evidence in the field of macrophage metabolism suggests that defining macrophage effector function by their metabolic pathways is likely to be an oversimplification [14]—underscoring the metabolic flexibility, and hence functional plasticity, characteristic of this cell type. Further in-depth investigation beyond the scope of the current study would be necessary to decipher which alternative energy pathways the GLUT1^ΔmΦ^ macrophages from our orthotopic-transplant tumor model are utilizing.

Of note, GLUT1^ΔmΦ^ mice were significantly protected from tumor growth compared with controls, an observation that was dependent on CTLs and NK cells. How do these macrophages promote anti-tumor lymphoid immunity? Our data showed that GLUT1^ΔmΦ^ macrophages enhanced their antigen presentation of OVA peptide to T cells. In line with this, it is likely that these GLUT1^ΔmΦ^ macrophages may also be better at presenting tumor antigens to CTLs, thus promoting increased anti-tumoral CTL effector functions. In addition, it is possible that the likely depletion of FA by GLUT1^ΔmΦ^ macrophages may result in an overall deficiency of either FA or glutamine, or both, in the tumor microenvironment alongside a surplus of glucose. This change in nutrient availability may encourage preferential utilization of glucose by infiltrating T and NK cells with a subsequent increase in effector function. The notion that nutrient availability can modify immune cell function is not without precedent. When highly glycolytic antigenic tumors outcompete T cells for glucose, this metabolic restriction has been shown to directly inhibit T cell effector function and promote tumor progression [61]. In addition, effector T_h_ cells, in direct contrast to T_Reg_ cells, have been shown to upregulate glycolysis and downregulate FAO [25,62]. CD4^+^ T cells specifically ablated of GLUT1 had impaired growth, proliferation, survival, and differentiation [63]. Taken together, these studies demonstrate the importance of glycolysis for T cell effector function. Thus, it is possible that infiltrating T cells and NK cells in orthotopic tumors of GLUT1^ΔmΦ^ mice respond to the excess glucose present in the environment by adopting glycolysis and subsequently acquire enhanced effector function to promote tumor clearance.

Given the significant tumor inhibition observed in GLUT1^ΔmΦ^ mice, we identified GLUT1 as a potential drug target. WZB117, a GLUT1-specific inhibitor, was also able to ameliorate disease, with similar upregulation of CTL and NK immunity. The reduction in tumor burden was not as prominent as observed in the GLUT1^ΔmΦ^ mice; however, this is not entirely surprising, as the genetic deletion approach resulted in a complete and whole-scale shutdown of glycolysis while WZB117, administered via the oral route in our experiments, may have transient effects. Alternatively, there may be tolerization of the drug or compensation with other GLUT isoforms over time. The tumor growth inhibition observed in our orthotopic tumor model with WZB117 is consistent with a report by Liu and colleagues demonstrating its anti-tumor effect in a variety of tumor cell lines and xenograft model [64]. In WZB117-treated animals, we noted an increase in expression of IL1β and other pro-inflammatory cytokines despite the attenuation of tumor burden, suggesting that the induction of anti-tumor CTL and NK immunity was sufficient to overcome this apparent increase in macrophage inflammation.

Importantly, the expression of GLUT1 on macrophages may also have prognostic value. In a comprehensive analysis of FFPE sections from multi-center PDAC patient cohorts using a multiplex, quantitative immunofluorescence staining approach, we show that the CD68+ GLUT1+ subset and CD68+ GLUT1+ HK2+ HIF1α+ subset are better predictors of survival in PDAC patients, compared with the individual metabolic markers alone. While others have shown that high HK2 [65], HIF1α [66], or GLUT1 [67,68] expression correlates with poor prognosis, we provide compelling evidence that the macrophage glycolytic signature is more closely linked to PDAC disease outcome. This signature could be used as a diagnostic tool to assist in predicting patient survival and as a patient stratification strategy for relevant pharmacologic modulation.

Some unanswered questions remain. Could signaling pathways such as mTOR and AMPK, critical nutrient sensors, and metabolic regulators [69,70,71], also be involved in the anti-tumor immune response, downstream of the metabolic shift in GLUT1^ΔmΦ^ macrophages? A recent report suggests that this is indeed possible. Induced AMPK deficiency in T-cell acute lymphoblastic leukemia (T-ALL) cells results in T-ALL death and abatement of disease in a syngeneic mouse model [40]. However, to address if the NLRP3, mTOR, or AMPK signaling play a role, proteins involved in these pathways would have to be specifically overexpressed or knocked down in macrophages from the orthotopic-transplant model, and in GLUT1^ΔmΦ^ macrophages, using genetic strategies. Detailed exploration of these signaling pathways in GLUT1^ΔmΦ^ mice via such complex approaches is beyond the scope of this current study.

It is known that the primary glucose transporter in macrophages is GLUT1 [19], as it is for T cells [63]. Given that there are at least 14 GLUT isoforms identified thus far [72], and that glycolysis is an essential fuel pathway, it is somewhat surprising that the knockdown of a singular isoform, GLUT1, permits a total shutdown of glycolysis without parallel compensation from other GLUT isoforms. This could be due to fixed post-translational processing in these cell types or flexibility in the consumption of alternative fuels. The reliance on GLUT1 in macrophages, therefore, makes it an ideal target for therapeutic intervention.

WZB117 is a specific inhibitor of GLUT1 and a number of inhibitors targeting metabolic enzymes are currently undergoing testing in cancer clinical trials [73]; although GLUT inhibitors have not yet passed the Phase II stage [72]. As GLUTs are expressed in both healthy and tumor cells, identification of a therapeutic window where macrophage and tumor cell glycolysis can be targeted specifically and in tandem, without bystander toxicity, is essential. 2-DG, an inhibitor of glycolysis, has been tested in mouse models of transplantation and systemic lupus erythematosus without apparent overt toxicity [74,75]. We were encouraged to note that in our study, WZB117-fed mice appeared to safely tolerate the drug at a 50mg/kg daily dose even after four weeks.

Ultimately, targeting immune cells provides an alternative approach to overcome the myriad mechanisms of tumor resistance, especially in the setting of PDAC. By not only eradicating the neoplastic cells but also rebalancing the perturbed immune equilibrium using interventions that target macrophage metabolism, we are optimistic that improved outcomes of this intractable disease can be achieved.

## 4. Materials and Methods

### 4.1. Mice

WT C57BL/6, GLUT1^fl/fl^; LysM-cre and associated “control” (LysM-cre alone, and/or GLUT1^fl/fl^ alone, whichever available) mice were housed and maintained under specific pathogen-free conditions in the Biological Resource Centre (BRC) of A*STAR, Singapore. GLUT1^fl/fl^ mice were a kind gift from Prof E. Dale Abel at the University of Iowa. All experiments were performed under the approval of the Institutional Animal Care and Use Committee (IACUC) of the BRC, according to the guidelines of the Agri-Food and Veterinary Authority (AVA) and the National Advisory Committee for Laboratory Animal Research (NACLAR) of Singapore (IACUC protocol #171230, approved to start 7 May 2017, renewed from #130879, approved to start 1 December 2013). The compounds KI20227 and WZB117 were custom-synthesized by GVK Biosciences. Both compounds were incorporated into standard rodent chow Harlan 2018 at 250 ppm and irradiated (Envigo–Teklad Diets, Indianapolis, IN, USA). In some experiments, gemcitabine (53J5044, Actavis, Parsippany, New Jersey, USA,) was administered via intraperitoneal (i.p.) injection at 50 mg/kg twice/week; anti-NK1.1 (Bio X Cell, PK136) or isotype control antibody (Bio X Cell, C1.18.4) was injected i.p. at 250 µg once/week; anti-CD8α (Bio X Cell, YTS169.4) or isotype control antibody (Bio X Cell, 2A3) was injected i.p. at 500 µg once/week.

### 4.2. Generation of Luciferase STable 1242L Cell Line

The luciferase gene was amplified from the PGL3 basic vector (E1751Promega, San Luis Obispo, CA, USA) and sub-cloned into the ITR-CAG-DEST-IRES-puro-ITR plasmid (kind gift from Marc Schmidt-Supprian). ITR-CAG-DEST-IRES-puro-ITR (Control plasmid) or ITR-CAG-Luciferase-IRES-puro-ITR (Luciferase) plasmid was mixed with SB100X transposase (1:1 ratio) and transiently transfected using Lipofectamine 2000 (11668027, Thermo Fisher Scientific, Waltham, MA, USA) into the FC1242 tumor cell line (kind gift from Dr. Dannielle Engle, Tuveson lab, Cold Spring Harbor Laboratory), derived from KRAS^G12D/+^; *Trp53^R172H/+^;* PDX-cre (KPC) mice ^27^. Three days post-transfection, transfected tumor cells were selected with 2 µg/mL puromycin for seven days. To validate the stable and constitutive expression of luciferase, cells were lysed and assayed for luciferase activity using the Luciferase Assay kit (E1483, Promega, San Luis Obispo, CA, USA). Transfected tumor cells were designated 1242L.

### 4.3. Generation of Orthotopic Pancreatic tumors

Orthotopic tumors were generated in WT C57BL/6 mice, aged 6–8 weeks. Tumor cells (1 × 10^5^) were resuspended in PBS and mixed with Matrigel (354230, BD Biosciences, San Jose, CA, USA) in a 1:1 ratio to a total volume of 50 µL, which was injected into the body of the pancreas through a laparotomy. Experimental groups were sex- and age-matched. Mice were euthanized at Day 27–35.

### 4.4. In Vivo Bioluminescent Imaging (BLI)

On Day 6, 13, 20, and 27 post tumor injection, mice were imaged using the IVIS Spectrum Imaging System (Perkin Elmer, Melbourne, Australia) to monitor tumor progression in vivo. Mice were first anesthetized with isoflurane and then injected intraperitoneally with 50 µL of D-Luciferin (5 mg/mL; Perkin Elmer). After 5 min, mice were positioned in the IVIS Spectrum and imaged at various exposures. Using LivingImage Software, the bioluminescence signal for each mouse was quantified as Total Flux (photons/second) using an exposure image where the signal was not saturated. BLI images shown in the figures were at 15 s exposure.

### 4.5. Tissue Dissociation

Tumors and pancreata were dissected from mice, finely minced with scissors in 15-mL scintillation vials, and washed once in cold PBS. Each pancreas was digested with 15 mL of 1 mg/mL collagenase IV (C5138, Sigma, St.Louis, MO, USA) and 0.5 mg/mL DNase I (Sigma, DN25) for 30 min at 37 °C. The pancreas was then dissociated through a 70-µm filter to obtain a single-cell suspension. CD45^+^ cells were pre-enriched using biotinylated anti-mouse CD45 antibody (103101, BioLegend, San Diego, CA, USA) with a Mouse Streptavidin RapidSpheres Isolation Kit (19860A, Stem Cell Technologies, Vancouver, BC, Canada). For tumor weight measurements, the entirety of the pancreas, with tumor attached, was weighed on a scale up to two decimal places.

### 4.6. Flow Cytometry

Single-cell suspensions of the dissociated and pre-enriched pancreata were resuspended in FACS buffer (10% fetal calf serum, 5% human serum, 0.01% sodium azide in PBS) with 0.3 mg/mL DNase I. After Fc blockade with anti-FcγRIII/II (BD Biosciences, San Jose, CA, USA), cells were stained with DAPI, and surface-stained or intracellularly (ICFC) stained with a combination of the antibodies shown in Table S1. For the ICFC, pancreas single-cell suspensions were stimulated with 100 ng/mL LPS (for myeloid IC panel), or with 10 ng/mL PMA and 1 µM ionomycin (for lymphoid IC panel) with Golgi Plug (555029, BD Biosciences, San Jose, CA, USA) for 4 h. Caspase 1 activity was measured using the Caspase-1-FAM-FLICA Assay (97ImmunoChemistry Technologies, Bloomington, MN, USA) according to the manufacturer’s instructions.

### 4.7. Seahorse Live Metabolic Flux Assay

The mitochondria oxygen consumption rate (OCR, O_2_ pmol/min) and extracellular acidification rate (ECAR, mpH/min) of macrophages were analyzed using an XF^e^96 extracellular flux analyzer (Seahorse Bioscience/Agilent Technologies, Lexington, MA, USA) according to the manufacturer’s instructions. Macrophages were harvested, washed, and resuspended in the experimental medium at 2 × 10^5^/well in flat-bottom 96-well Seahorse microculture plates, and incubated in a non-CO_2_ incubator for 1 h at 37 °C prior to the start of each assay. For the *Glycolysis Stress Test* ECAR analysis, macrophages were resuspended in ECAR medium (DMEM base (no bicarbonate) with 2 mM L-glutamine, 143 mM NaCl, and 0.5% phenol red (pH 7.35)). This test consisted of four consecutive stages: basal (without drugs), glycolysis induction (10 mM glucose), maximal glycolysis induction (5 µM oligomycin), and glycolysis inhibition (100 mM 2DG). Glycolytic capacity was calculated as follows: (mean of highest ECAR values induced by glucose or oligomycin)—(mean of basal ECAR values).

### 4.8. Metabolomics Analysis

For metabolite extraction, cells were collected by centrifugation and washed twice in 5% mannitol solution (10 mL volume and then 2 mL). For extracting ionic metabolites, cells were treated with 800 µL of methanol and left to rest for 30 s to inactivate enzymes. The cell extract was then treated with 550 µL of Milli-Q water containing internal standards (H3304-1002, Human Metabolome Technologies (HMT), Boston, MA, USA), and left to rest for another 30 s. The extract was centrifuged at 2300× *g* for 5 min at 4 °C and then 800 µL of upper aqueous layer was applied to a Millipore 5-kDa cutoff filter (UltrafreeMC-PLHCC, HMT, Boston, MA, USA) to remove macromolecules (9100× *g*, 4 °C, 2 h). The filtrate was re-suspended in 50 µL of Milli-Q water for metabolome analysis by HMT. For non-polar metabolites, washed cells were treated with 1 mL ethanol containing the internal standards and then with 1 mL Milli-Q water. The cell solution was sonicated for 5 min and centrifuged at 2700× *g* for 5 min at 4 °C. The supernatant was dried under nitrogen purge and re-suspended in 100 µL of 50% isopropanol (*v/v*) for metabolome analysis by HMT.

Metabolomics analysis was conducted with the Dual Scan package of HMT using capillary electrophoresis time-of-flight mass spectrometry (CE-TOFMS) and liquid chromatography time-of-flight mass spectrometry (LC-TOFMS) based on methods described previously [76,77]. Briefly, CE-TOFMS analysis was carried out using an Agilent CE capillary electrophoresis system equipped with an Agilent 6210 time-of-flight mass spectrometer (Agilent Technologies, Waldbronn, Germany). LC-TOFMS analysis was carried out by Agilent 1200 HPLC pump with an Agilent 6210 time-of-flight mass spectrometer (Agilent Technologies). The systems were controlled by Agilent G2201AA ChemStation software version B.03.01 for CE (Agilent Technologies) and MassHunter for LC (Agilent Technologies). The spectrometer was scanned from *m/z* 50 to 1000, and peaks were extracted using MasterHands automatic integration software (Keio University) to obtain peak information, including *m/z*, peak area, and migration time (MT) [78]. Signal peaks corresponding to isotopomers, adduct ions, and other product ions of known metabolites were excluded, and remaining peaks were annotated according to the HMT metabolite database based on their *m*/*z* values with the MTs. Areas of the annotated peaks were then normalized based on internal standard levels and sample amounts to obtain relative levels of each metabolite. Hierarchical cluster analysis (HCA) and principal component analysis (PCA) were performed by HMT’s proprietary software, PeakStat, and SampleStat, respectively. Detected metabolites were plotted on metabolic pathway maps using VANTED software [79].

### 4.9. Western Blot

Cell lysates from sorted macrophages were prepared for Western blot. Samples with equal amounts of protein (10 µg) were resolved on 15% acrylamide denaturing gels (SDS-PAGE). Proteins were transferred to nitrocellulose membranes (Trans-Blot Turbo Mini Nitrocellulose Transfer Packs, Bio-Rad, Billerica, MA, USA) using the Bio-Rad Trans-Blot Turbo Transfer System for 7 min. The membrane was blocked in 1× Tris buffered saline and 0.1% Tween 20 (TBS-T) containing 5% low-fat milk for 1 h and probed overnight at 4 °C with primary antibodies diluted in blocking buffer. Primary antibodies used were: β-actin 1:500 (MAB1501, Merck Millipore, Burlington, MA, USA), IL1β 1:250 (sc-7884, Santa-Cruz Biotechnology, Santa Cruz, CA, USA) to detect pro-IL1β and IL1β, and caspase-1 1:500 (Santa-Cruz Biotechnology, sc-56036) to detect pro-caspase-1 and caspase-1, followed by anti-mouse 1:5000 (Abcam, ab97023) and anti-rabbit 1:5000 (Abcam 97051) HRP-conjugated secondary antibodies. The immunoreaction was detected with SuperSignal™ West Pico Chemiluminescent Substrate (ThermoFisher Scientific, Waltham, MA, USA) and visualized using BioRad ChemiDoc XRS system.

### 4.10. RT-qPCR

Cells were lysed with Qiazol (Qiagen), and total RNA was purified using the RNeasy Mini kit (Qiagen, Germantown, Maryland, USA). DNase-treated total RNA (0.5–1 µg) was reverse-transcribed to cDNA using iScript Reverse Transcription Supermix (Bio-Rad). Expression of GLUT1, *HK1*, *HK2, GPI, PFKB1, ALDOA, PGK, PKM2, LDHA, HIF1α, CPT1A, CPT1B, ACADL, ACADM, ACC, FASN, PDK1, CS, IDH1, SDHB, FH1, MDH1, PGC1β, STAT6,* and *β-actin* were determined by quantitative PCR with KAPA Sybr FAST ABI Prism 2× qPCR Master Mix (KAPA Biosystems, Wilmington, MA, USA) using an ABI 7900HT Fast Real-Time PCR System (Applied Biosystems). Levels of the housekeeping gene *β-actin* were also measured and used to normalize the data. The list of qPCR primer sequences is listed in Table S2.

### 4.11. Immunofluorescence of ASC

Sorted PEC macrophages were seeded at 2.5 × 10^5^ cells/200 µL of complete media per well of an 8-well Ibidi µ-slide. Cells were stimulated with or without 1 µg/mL of LPS for 3 h at 37 °C, with 10 µM of nigericin added for a further 30 min. After stimulation, cells were fixed with 4% PFA for 30 min at 37 °C, washed twice with PBS, and permeabilized with Perm/Block buffer (10% goat serum, 1% FBS, 0.5% Triton X-100 in PBS) for 30 min at 37 °C. Cells were then stained with 10 µg/mL of anti-ASC mouse monoclonal antibody (04-147, Millipore, Burlington, MA, USA) overnight at 4 °C, washed, and stained with 1:1000 with donkey anti-mouse Alexa Fluor-488 secondary antibody (A21202, Invitrogen/Thermofisher Scientific, Waltham, MA, USA) for 1 h at room temperature. After washing, cells were counterstained with DAPI to visualize nuclei and imaged at 100× magnification using an FV-1000 confocal system with an inverted Olympus IX81 microscope. At least 10 Z-stacks of 1 µm were combined to form the final images shown in Figure 3.

### 4.12. In Vitro OTI T Cells Stimulation

Naïve splenocytes isolated from OTI mice were primed with OVA257-264 peptide and recombinant human IL-2 (212-IL, R&D Systems, Minneapolis, MN, USA) in DMEM medium supplemented with 10% FBS, 2 mM L-glutamine, 100U/mL penicillin/streptomycin, and 50mM β-mercaptoethanol for 24 h. OTI T cells were further expanded for at least two days in the presence of recombinant human IL-2. For antigen-restricted T cell stimulation assay, FACS-sorted PEC macrophages were pulsed with OVA peptide for 20 min, washed twice to remove free peptide, and then co-cultured with the expanded OTI T cells using a U-bottom, 96-well plate for 6 h in the presence of Golgi Plug (BD Biosciences 555029). Activation of OTI cells was determined by intracellular IFNγ staining through flow cytometry.

### 4.13. Publicly Available PDAC Data (TCGA and QCMG Cohorts)

Selected gene expression and clinical survival data were obtained from cBioPortal (http://www.cbioportal.org/, accessed on 20 May 2012) for the QCMG and TCGA (https://cancergenome.nih.gov/, accessed on 20 May 2012) data sets. The gene expression data were thresholded at ten-percentile intervals for each gene and used in a series of log-rank survival tests. The percentiles showing the largest differences in the survival curves were selected for interpretation and further analyses. All analysis and visualizations were done using the R statistical language (v3.3.1, R studio, Boston, MA, USA).

### 4.14. Multiplex Quantitative Immunofluorescence (MQIF) Staining and Analysis

Three PDAC patient cohorts from the Stanford Medical Center, National Cancer Centre Singapore (SingHealth CIRB 2012/879/B), and National Cancer Institute Singapore (DSRB 2012/00939) were obtained. The collection of Singaporean patient sections was approved by the Institutional Review Board of Singapore. PDAC human tissue FFPE sections were evaluated and confirmed of diagnosis by board-certified pathologists at each of these 3 institutions for analysis. PDAC human tissue FFPE sections (adjacent “normal” = 6; patient = 50) were stained in a 6-plex staining protocol (Perkin Elmer, Melbourne, Australia) in this sequence: First cycle—primary antibody anti-CD68 1:150 (Dako, PG-M1), followed by secondary antibody anti-mouse HRP (Dako, K4001), and then TSA-Cy3 1:100 (Perkin Elmer Life Sciences, FP1170), with microwave treatment using citrate pH 6 Antigen Retrieval Buffer (00-4955-58, eBioscience, San Diego, CA, USA). Second cycle—primary antibody anti-GLUT1 1:250 (Thermo Fisher Scientific, RB-9052-P1), followed by secondary antibody anti-rabbit HRP (Dako, K4003), and then TSA-Cy3.5 1:100 (Perkin Elmer Life Sciences, FP1484), with microwave treatment using citrate pH 6 Antigen Retrieval Buffer (eBioscience, 00-4955-58). Third cycle—primary antibody used was anti-CD163 1:200 (ab188571, Abcam, Cambridge, MA, USA), followed by secondary antibody anti-rabbit HRP (Dako, K4003), and then TSA-Cy5.5 1:100 (Perkin Elmer Life Sciences, FP1486), with microwave treatment using citrate pH 6 Antigen Retrieval Buffer (eBioscience, 00-4955-58). Fourth cycle—primary antibody used was anti-HK2 1:150 (Abcam, 104836), followed by secondary antibody anti-mouse HRP (K4001, Dako, Carpinteria, CA, USA), and then TSA-FITC 1:100 (Perkin Elmer Life Sciences, FP1168), with microwave treatment using Tris pH 9 (Dako, S2367). Fifth cycle—primary antibody used was anti-HIF1α 1:100 (Novus Biologicals, NB100-105), followed by secondary antibody anti-mouse HRP (Dako, K4001), and then TSA-Cy5 1:100 (Perkin Elmer Life Sciences, FP1171), with microwave treatment using citrate pH 6 Antigen Retrieval Buffer (eBioscience, 00-4955-58). After the fifth cycle, the sections were counterstained with DAPI (Sigma Life Science, D9542-5MG).

At least 15 fields of view were imaged per patient section using the Mantra Workstation (Perkin Elmer). Objective signal counts, tumor/stroma compartments, and cell phenotypes were defined and quantified in these stained sections using multispectral fluorescence analysis and spectral unmixing with the InForm software (PerkinElmer). Inform was trained by board-certified pathologists in Singapore to recognize tumor versus the stroma compartment on each stained section, based on tissue structures, morphology, size, and characteristics of nuclei. Subsequently, all analysis was based on the “Entire Cell Total Normalized Counts” generated by the Inform software. Using in-house codes, we studied the association of a cell subset (for e.g., CD68+GLUT1+) to survival and stage progression, by computing the percentage composition of the cell subset on the basis of individual patients. Cells captured from all the tissue sections of a patient were assigned to binary cell subsets, the target, and the complement (CD68+GLUT1- in this example) subsets.

Survival analysis on manually selected cell subsets was analyzed using the nearest-template prediction (NTP) method. Subsets that were confounded on either gender or ethnicity were excluded and the remaining subsets were then thresholded at ten-percentile intervals and used in a series of log-rank survival tests. The percentiles showing the largest differences in the survival curves were selected for interpretation and further analyses. All analysis and visualizations were done using the R statistical language (v3.3.1).

### 4.15. Statistics

Data are presented as means ± s.e.m. Statistical significance was determined by the Student’s t-test (two-tailed), *p* values of < 0.05 were considered significant. Survival curves in mice and patients were measured using the Kaplan–Meier method, and significance was determined using the log-rank test. For the MQIF of PDAC patient cohorts’, a Kruskal–Wallis rank sum test was first performed to examine if the composition of a cell subset is significantly different in any stage. If there was no difference in cell subset composition across all the stages of PDAC, a Mann–Whitney test was used to check if there was a significant difference between normal and PDAC patients. For the survival curves of the PDAC patient cohorts, a Kruskal–Wallis rank sum test was used to determine if any difference found in subset composition was due to confounding factors such as gender or ethnicity. Multiple testing corrections for the cell subsets were done using the Benjamini & Hochberg method. The risk of poor survival in each subset was quantified by hazard risk ratio derived from cox hazard regression analysis.

## Data Availability

Selected gene expression and clinical survival data were obtained from cBioPortal (http://www.cbioportal.org/, accessed on 20 May 2012) for the QCMG and TCGA (https://cancergenome.nih.gov/, accessed on 20 May 2012) data sets.

## Supplementary Materials

The following are available online at https://www.mdpi.com/article/10.3390/ijms22126350/s1.
