# Supplementary material for: Targeting Glycolysis in Macrophages Confers Protection Against Pancreatic Ductal Adenocarcinoma
Source: Int J Mol Sci. 2021 Jun 14;22(12):6350. doi: 10.3390/ijms22126350 (PMC8231859; doi:10.3390/ijms22126350)
Supplement: Supplementary file 1 [file ijms-22-06350-s001.zip › ijms-1224135-SI.pdf]

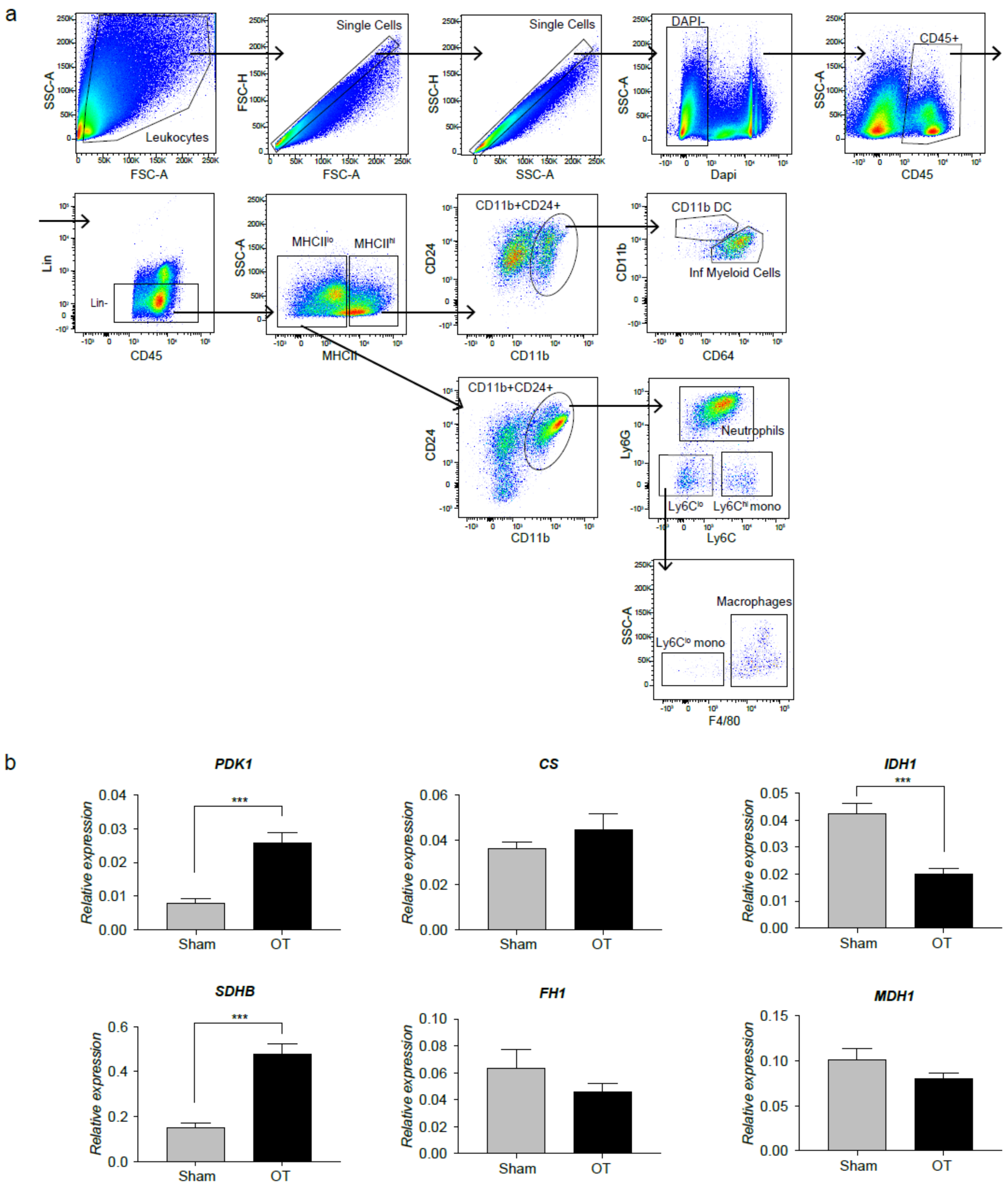

Supplementary Figure S1. Related to Figure 1. (**a**) Flow cytometry gating of different myeloid cell populations in a representative OT pancreas. (**b**) PEC macro-phages from OT and sham controls were assessed at the transcript level for PDK1, CS, IDH1, SDHB, FH1, MDH1 (n = 3 mice pooled per group, three indepen-dent experiments). Data are means ± s.e.m., ***$p < 0.001$ by unpaired student's t-test with 95% confidence interval.

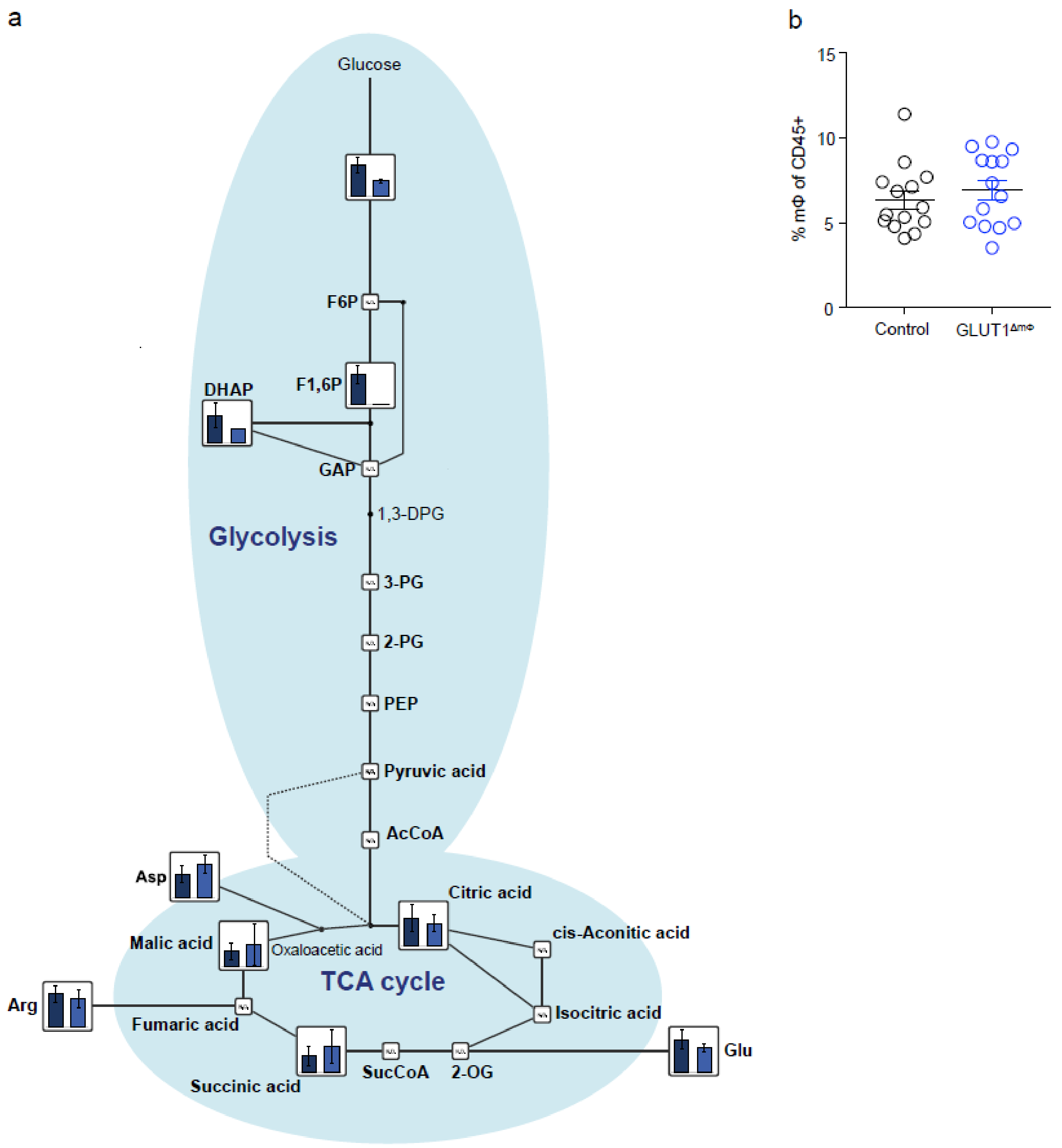

Supplementary Figure S2. Related to Figure 2. (**a**) Glycolysis pathway and TCA cycle analysis generated from HMT Dual Scan (CE-MS and LC-MS) metabolome profiling. Bar graph inset of pathways show the ratio of relative area under mass peak of the individual metabolites between control (black) and GLUT1ΔmΦ (blue) PEC macrophages (control, n = 3; GLUT1ΔmΦ, n = 4, n = 3 mice pooled per sample. (**b**) Scatter plots of % pancreatic macrophages of total CD45+ cells (control, n = 14; GLUT1ΔmΦ, n = 14, pooled from three independent experiments).

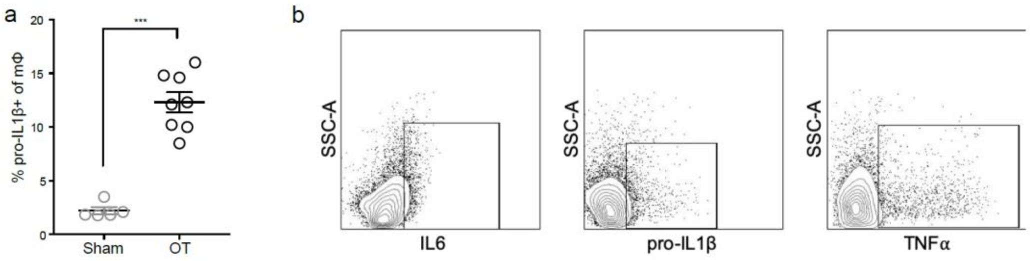

Supplementary Figure S3. Related to Figure 3. (a) Scatter plots of the % pro-IL1β+ of total pancreatic macrophages (sham, n = 5; GLUT1ΔmΦ, n = 8, pooled from two independent experiments). Data are means ± s.e.m. ***p < 0.001 by unpaired student's t-test with 95% confidence interval. (b) Representative flow plots of intracellular staining by flow cytometry shown for IL6, pro-IL1β, TNFa.

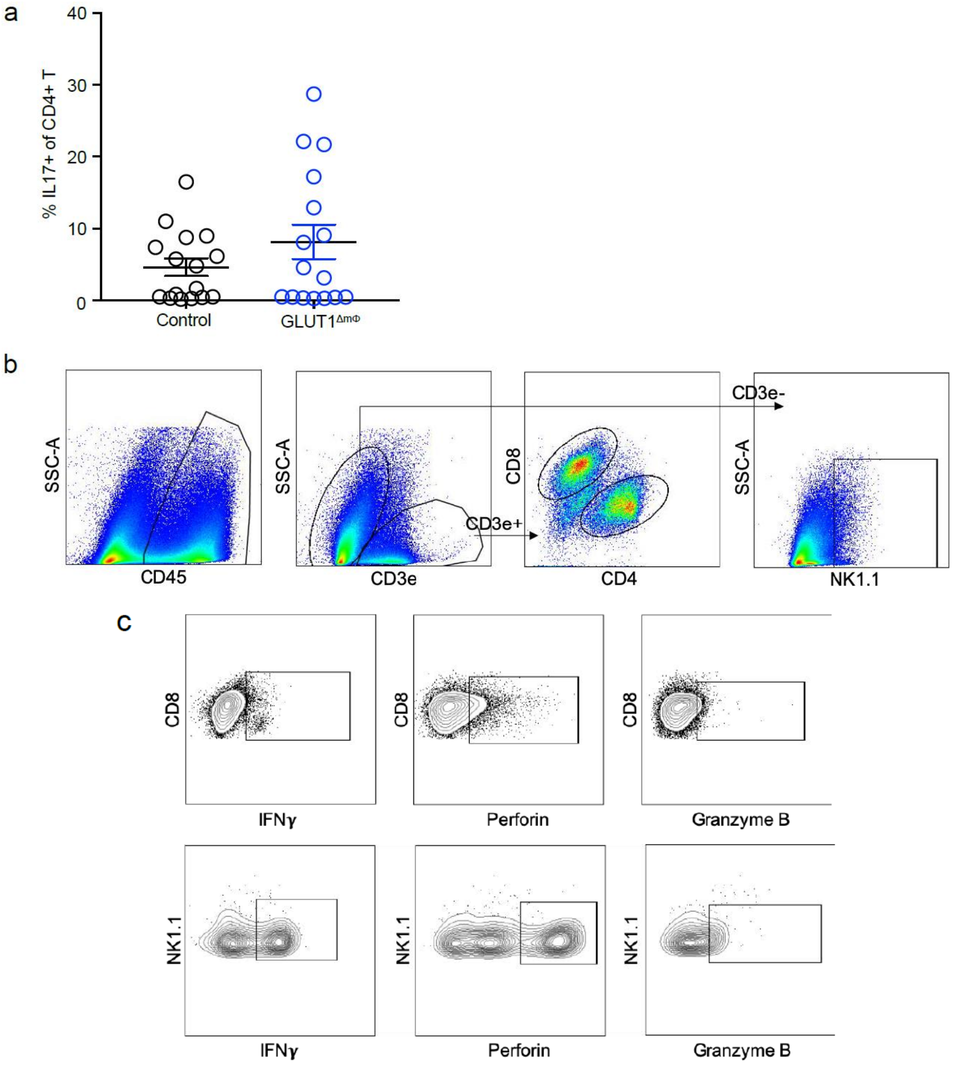

Supplementary Figure S4. Related to Figure 4. (**a**) Scatter plot of % IL17+ of total CD4+ cells in the pancreata of control (black) and GLUT1ΔmΦ (blue) mice (n = 16 mice per group, pooled from three independent experiments). (**b**) Flow cytometry gating strategy for CD8+ and NK cells. (**c**) Representative flow plots of intracellular staining by flow cytometry shown for IFNg, perforin and Granzyme B.

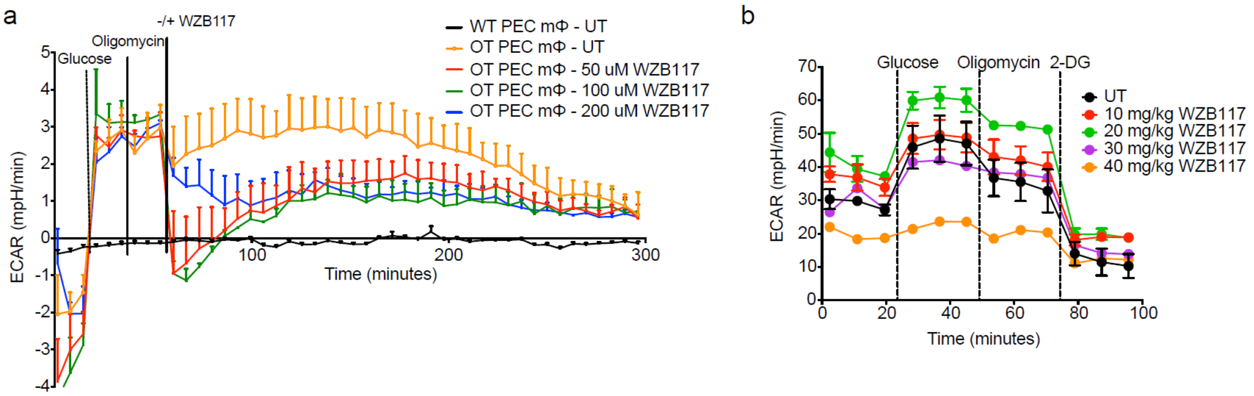

Supplementary Figure S5. (**a**) Seahorse trace of a modified glycolysis stress test on WT and OT PEC macrophages where ECAR was detected up to the 300th minute after start of assay. In lieu of 2-DG, varying concentrations of WZB117 was injected in Port C. Representative trace shown of two independent experiments. (**b**) Mice bearing 2-week old orthotopic tumors were injected daily for 7 days i.p. with varying concentrations of WZB117. Shown is the Seahorse trace of a glycolysis stress test on ex vivo sorted PEC macrophages from these mice. Representative trace shown of two independent experiments.

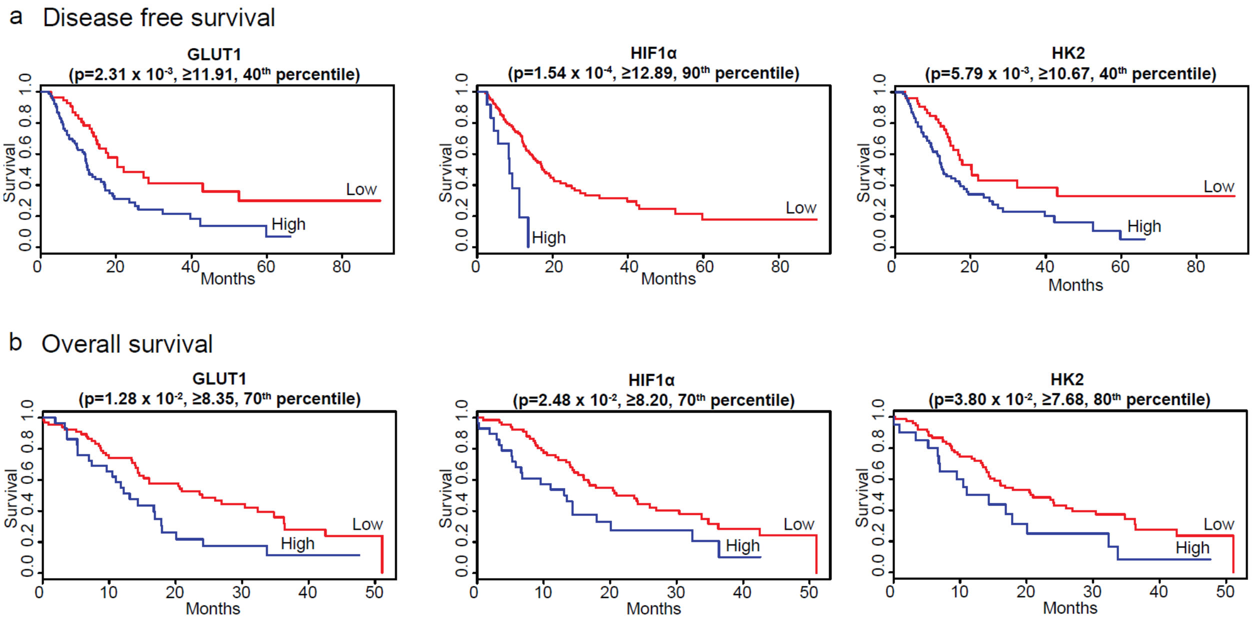

Supplementary Figure S6. Gene expression and clinical survival data was extracted from the TCGA and QCMG cohorts, and analyzed the same way as described in Figure 6. Shown are Kaplan-Meier disease-free survival curves for the TCGA cohort (**a**) and overall survival curves for the QCMG cohort (**b**) of GLUT1, HIF1α and HK2 for which the percentile cut-offs yielded significant difference between the high and low patient populations.

Supplementary Table S1. List of mouse antibodies used for flow cytometry.

Antibody	Company Catalog Number
**Myeloid Panel**	
CD11b-Brilliant Violet 650	BioLegend 101239
MHCII-Alexa 700	BioLegend 107622
CD45-PerCP	BioLegend 103130
NK1.1-FITC	BD Biosciences 108706
CD3-FITC	BD Biosciences 553062
CD19-FITC	BD Biosciences 115506
Ly6G-APC	BioLegend 127614
Ly6C-APC Cy7	BioLegend 128026
CD24-Pacific Blue	BioLegend 101820
CD11c-PE Cy7	BioLegend 117318
CD64-PE	BioLegend 139304
F4/80-PE Texas Red	BioLegend 123146
**Lymphoid Panel**	
CD62L-Alexa700	BioLegend 104426
CD4-Brilliant Violet 605	BioLegend 100547
CD8a-eFluor 450	eBioscience 48-0081-82
CD3e-APC	BioLegend 100312
CD25-PE Cy7	BioLegend 102016
CD19-PE CF594	BD Biosciences 562291
CD45-BUV 395	BD Biosciences 564279
NK1.1-FITC	BD Biosciences 108706
Pan γδ-PE	eBioscience 12-5711-82
CD90.2-APC Cy7	BioLegend 105328
CD44-PerCP Cy5.5	BioLegend 103032
**Myeloid Intracellular Cytokine Panel**	
Ly6G-Alexa450	BioLegend 127612
F4/80-PE Cy7	BioLegend 123113
CD11b-Brilliant Violet 650	BioLegend 101239
CD45-Brilliant Violet 295	BD Biosciences 564279
IL-6-PerCP Cy5.5	eBioscience 46-7061-80
IL-12p40-FITC	BioLegend 580564
TNFα-PE	eBioscience 12-7321-82
IL-1β(pro)-APC eFluor780(Cy7)	eBioscience 47-7114-82
**Lymphoid Intracellular Cytokine Panel**	
CD4-Brilliant Violet 605	BioLegend 100547
CD8-PE Cy7	eBioscience 25-0081-81
CD3-APC	BioLegend 100312
CD45-BUV395	BD Biosciences 564279
NK1.1-APC Cy7	BioLegend 108724
Pan γδ-PE Texas Red	BD Biosciences 563532
IFNγ-BV650	BD Biosciences 563854
Granzyme B-FITC	BioLegend 11-8398-82
Perforin-PE	eBioscience 12-9392-82
IL-17-eFluor 45	eBioscience 48-7177-82
**T cell Activation Panel**	
CD45-BUV395	BD Biosciences 564279
CD4-Brilliant Violet 605	BioLegend 100547
CD8-PE Cy7	eBioscience 25-0081-81
CD3-APC	BioLegend 100312
NK1.1-FITC	BD Biosciences 108706
CD69-Pacific Blue	Biolegend 104523
**Pancreas Sorting Panel**	
CD11b-Brilliant Violet 650	BioLegend 101239
CD45-PerCP	BioLegend 103130
CD3-Pe Cy7	BioLegend 100220
NK1.1-FITC	BD Biosciences 108706
CD19-FITC	BD Biosciences 115506
Ly6G-APC	BioLegend 127614
Ly6C-APC Cy7	BioLegend 128026
F4/80-PE	eBioscience 12-4801-82
**PEC Sorting Panel**	
CD11b-Brilliant Violet 650	BioLegend 101239
CD45-PerCP	BioLegend 103130
F4/80-PE	eBioscience 12-4801-82

Supplementary Table S2. List of Mouse primers used for RT-qPCR.

	Forward 5'-3'	Reverse 5'-3'
**GLUT1**	CATCCTTATTGCCCAGGTGTTT	GAAGACGACACTGAGCAGCAGA
**HK1**	TGCCATGCGGCTCTCTGATG	CTTGACGGAGGCCGTTGGGTT
**HK2**	AGCTGTTTGACCACATTGCC	CACGCCACTGGACTTGAAC
**GPI**	GGGACCCCTCATGGTGACT	GTCTTGGAGGCGATTATAAAGAGG
**PFKB1**	GCCACCTGTCCTACATCAAGA	TTCACTCTCACCATGTCGGC
**ALDOA**	AGAACACCGAGGAGAACAGG	AGTTGTCTCGCCATTGGTTC
**PGK**	GGAAGCGGGTCGTGATGA	GCCTTGATCCTTTGGTTGTTTG
**PKM2**	CCCTCGGGCTCCTATCATTG	TCCATGGCCAAGTTTACACGA
**LDHA**	GTGTAACTGCGAACTCCAAGC	TGGATTGGAGACGATCAGCAG
**HIF1α**	GTCGGACAGCCTCACCAAACAG	TAGGTAGTGAGCCACCAGTGTCC
**CPT1A**	TGCACTACGGAGTCCTGCA	GGACAACCTCCATGGCTCA
**CPT1B**	GTCGCTTCTTCAAGGTCTGG	AAGAAAGCAGCACGTTCGAT
**ACADL**	AGGTGTTCATCACTAATGGCTGG	TCCCATCTTATGCAGCTTCCG
**ACADM**	TGAAGGTTGAACTCGCTAGGC	CTCCGAAAATCTGCACGGC
**ACC**	CTGGCTGCATCCATTATGTCA	TGGTAGACTGCCCGTGTGAA
**FASN**	AGGGACCCTACCGCATAGCTG	GCCCGGAGCTTGTGGTAGAAG
**PDK1**	GACTGTGAAGATGAGTGACCG	CAATCCGTAACCAAACCCAG
**CS**	CCCAGGATACGGTCATGCAG	CTGTGAGCGTCTACGTTGGG
**IDH1**	ATGCAAGGAGATGAAATGACACG	GCATCACGATTCTCTATGCCTAA
**SDHB**	AAGAAGGATGAGTCCCAGGAG	CTTGTCTCCGTTCCACCAGTA
**FH1**	GGTGGTTTGGCAGACTGG	AGCCTCGCTGAGTGCAAC
**MDH1**	AACGGACAAAGAAGAGATTGCC	GCGTATTTCTCCAAGGCTGTG
**PGC1b**	ACTATGATCCCACGTCTGAAGAGTC	CCTTGTCTGAGGTATTGAGGTATTC
**STAT6**	CTGGGGTGGTTTCCTCTTG	TGCCCGGTCTCACCTAACTA
**β-actin**	AGAGGGAAATCGTGCGTGAC	CAATAGTGATGACCTGGCCGT

Supplementary Table S3. Cox hazard regression analysis of different subsets on PDAC survival.

Parameter	Percentile	Threshold (%)	Hazard Ratio (HR)	*p* value
All_Glut1+	80	59.85	2.56	3.78×10^{-2}
All_Glut1-	20	40.15	0.39	
CD68+Glut1+	70	67.00	3.75	1.96×10^{-3}
CD68+Glut1-	30	33.00	0.27	
CD68+HK2+Glut1+HIF1a+	60	27.49	3.49	2.32×10^{-3}
Except_CD68+HK2+Glut1+HIF1a+	40	72.51	0.29	
